# Serum ACE2 activity as a novel biomarker of assessment of severe aortic stenosis

**DOI:** 10.1007/s11357-025-01792-6

**Published:** 2025-07-15

**Authors:** Anita Kurczina, Arnold Péter Ráduly, Ivetta Mányiné Siket, Zsófia Pólik, Bertalan Kracskó, Attila Béla Kertész, Ágnes Balogh, Andrea Molnár, Tibor Fülöp, Laura Antal, Csaba Ötvös, Miklós Fagyas, Tamás Szerafin, Attila Tóth, Zoltán Papp, Zoltán Csanádi, Attila Borbély

**Affiliations:** 1 Division of Cardiology, Department of Cardiology, Faculty of Medicine, University of Debrecen, Móricz Zsigmond str. 22., H-4032 Debrecen, Hungary; 2 Division of Clinical Physiology, Department of Cardiology, Faculty of Medicine, University of Debrecen, Debrecen, Hungary; 3 Kálmán Laki Doctoral School, University of Debrecen, Debrecen, Hungary; 4 Department of Surgery, Faculty of Medicine, University of Debrecen, Debrecen, Hungary; 5 Division of Cardiac Surgery, Department of Cardiology, Faculty of Medicine, University of Debrecen, Debrecen, Hungary

**Keywords:** sACE2, Biomarkers, Aortic stenosis, Left ventricular function, NT-proBNP

## Abstract

Aortic stenosis (AS) is the most prevalent valve disease in developed countries, with its incidence rising in the aging population. The current criteria for aortic valve replacement (AVR) are based on subjective symptoms and left ventricular ejection fraction (LVEF), which may not adequately reflect left ventricular (LV) dysfunction. This highlights the necessity for objective biomarkers to evaluate subclinical LV dysfunction. Serum angiotensin-converting enzyme 2 (sACE2) has emerged as a promising novel biomarker for cardiovascular diseases. To investigate the association between sACE2 activity and different flow-grade categories of severe AS, compare it with the traditional biomarker N-terminal pro-brain natriuretic peptide (NT-proBNP), and assess the utility of sACE2 as a biomarker for AS. sACE2 and NT-proBNP were measured in 175 patients (97 male, 78 female, mean age 75 ± 8 years) diagnosed with severe AS (aortic valve area, AVA ≤ 1 cm^2^). Patients were classified into 5 groups depending on LV flow state and pressure gradient levels: normal flow-low gradient (NF-LG), normal flow-high gradient (NF-HG), low flow-high gradient (LF-HG), low flow-low gradient (LF-LG), and paradoxical low flow-low gradient (PLF-LG) AS. Both biomarkers showed a general increase with advanced stages of severe AS (NF-LG: 65.0 ± 3.5 U/ml; LF-LG: 148.1 ± 16.8 U/ml; *P* < 0.05 for sACE2 and NF-LG: 687 ± 193 pg/ml; LF-LG: 5966 ± 1076 pg/ml; *P* < 0.05 for NT-proBNP). Notably, PLF-LG patients exhibited NT-proBNP levels similar to NF groups (PLF-LG: 1010 ± 218 pg/ml). Both biomarkers negatively correlated with LVEF and AVA. Receiver operating characteristic (ROC) analysis revealed that sACE2 provides incremental value over NT-proBNP in detecting subclinical LV dysfunction, with a 44% specificity for sACE2 compared to 6% for NT-proBNP at 98,67% sensitivity. The assessment of sACE2 activity in patients with AS provides valuable insights into disease stage and progression, supporting clinical decision-making and optimizing the timing of AVR. Furthermore, sACE2 activity serves as a moderately sensitive blood biomarker for identifying patients at risk of AS.

## Introduction

Aortic stenosis (AS) is the most common valve disease in developed societies, and its prevalence increases with age sharply [1]. In the coming decades, as the population ages, the prevalence of AS is expected to increase, which will put an even greater strain on healthcare resources and society as a whole [2].

Current international recommendations [3, 4] for the echocardiographic evaluation of patients with AS depend on measurement of mean pressure gradient (P_mean_), peak transvalvular velocity (V_max_), and valve area (AVA). Under the same terminology of severe (aortic valve area, AVA ≤ 1cm^2^) we can identify haemodynamically different entities in terms of transvalvular flow (normal or low) rates and pressure gradients (high or low) development. These flow-grade patterns suggest that they are likely at a more advanced stage of their disease [5]. The only treatment option for severe AS is the prosthetic aortic valve replacement (AVR), which timing is currently decided based on symptomatic status and left ventricular ejection fraction (LVEF). However, the evaluation of AS-related symptoms can be challenging [6] and LVEF is not a sensitive marker of subclinical LV dysfunction [7]. Hence, these may potentially lead to inappropriate delay of AVR causing dramatic increase in mortality [8].

The use of biomarkers as indicators of pathogenic processes has increased significantly in the last few decades. Biomarkers reflecting maladaptive myocardial structural changes, such as wall stress, fibrosis, and myocyte death, can serve as objective, examiner-independent indicators of LV remodeling and impending myocardial decompensation [9, 10]. Current guidelines for risk stratification recommend the measurement of natriuretic peptides (i.e. NT-proBNP) in asymptomatic patients to guide treatment decisions and the management of AS [11].

The discovery of the membrane-bound ACE2 enzyme in 2000 [12], expressed in various cell types [13], has expanded our knowledge about the renin-angiotensin system. As a counter-regulatory pathway with degradation of angiotensin II [12], the ACE2 emerged as a regulator of cardiovascular biology [14]. Activity of soluble form (sACE2) is increased in the presence of cardiovascular diseases (CVDs) and predicts adverse events as a novel biomarker [15–20].

Our study aimed to investigate sACE2 activity throughout the natural history of severe AS parallel with the deterioration of haemodynamic parameters across different flow-grade categories. We hypothesized that sACE2 activity positively correlates with worsening echocardiographic parameters and serves as an indicator of adverse myocardial changes, transitioning from AS with compensated LV function to decompensated EF. Additionaly, we examined changes in NT-proBNP levels, the most reliable and widely used biomarker for AS [21].

## Methods

The study protocol was approved by the Regional and Institutional Ethics Committee, University of Debrecen (MTA AHT-T/281734) and by the Medical Research Council of Hungary. The research complied with the principles outlined in the Declaraion of Helsinki. All patients provided written inform consent to the study.

### Study population

We prospectively included consecutive patients with asymptomatic or symptomatic severe AS, defined as AVA ≤ 1 cm^2^ or AVAi < 0,6 cm/m^2^, admitted for preoperative evaluation or valve intervention to our clinic. Patients with more than moderate concomitant valvular heart disease were excluded from the study. The final population studied consisted of 175 patients from whom a venous blood sample was collected for the measurement of sACE2 activity and NT-proBNP levels. The collection of baseline echocardiographic, demographic and clinical data was obtained from medical documents. The comprehensive transthoracic echocardiography was performed by experienced echocardiographers using commercially available ultrasound systems.

### AS grading classification

Depending on LV flow state and pressure gradient level (ie, LVEF, P_mean_, stroke volume index [SVi]) patients were classified in 5 clinically relevant groups: normal-flow, low-gradient (NF-LG, LVEF ≥ 50%, P_mean_ < 40 Hgmm), normal-flow, high-gradient (NF-HG, LVEF ≥ 50%, P_mean_ ≥ 40 Hgmm), low-flow, high-gradient (LF-HG, LVEF < 50%, P_mean_ ≤ 40 Hgmm), low-flow, low-gradient (LF-LG, LVEF < 50%, P_mean_ < 40 Hgmm) and paradoxical low-flow, low-gradient (PLF-LG, LVEF ≥ 50%, P_mean_ < 40 Hgmm, SVi ≤ 35 mL/m^2^).

### Blood sampling and measurement of the serum ACE2 activity

At patient recruitment, blood samples were collected into native tubes using standard aseptic techniques and incubated for 60 min at room temperature. After clotting was completed, serum was separated by centrifugation (1,500* g*, 15 min) and stored at −20 °C until measurement of sACE2 activity, which was performed using a quenched fluorescence substrate-based assay as described previously with some modifications [15, 16, 22]. The reaction mixture (200 µl) contained 20 µl serum, 80 µl assay buffer (500 mM NaCI, 100 µM ZnCI_2_, 75 mM TRIS HCI, pH 6.5) and 100 µl ACE2-specific fluorescent substrate (7-methoxycoumarin-4-yl)acetyl-Ala-Pro-Lys(2,4-dinitrophenyl)-OH [Mca-APK(Dnp) (50 µM)] (EZ Biolab, Carmel, USA). All chemicals were from Sigma (St. Louis, MO, USA) if not stated otherwise.

### Statistical analysis

Statistical analysis was performed using GraphPad Prism, version 6.0 (GraphPad Software, Inc., San Diego, CA, USA). Descriptive analysis was presented as mean ± SD, n (%), or median ± IQR to compare the baseline characteristics between patients in all groups (Table 1). sACE2 activity and NT-proBNP levels in different groups did not pass the Kolmogorov–Smirnov normality test, therefore nonparametric evaluations were used. Kruskal–Wallis test with Dunn’s multiple comparisons test was performed for the multiple groups. Correlations were evaluated by calculating the Spearman’s rank correlation coefficient between echocardiographic parameters and biomarkers. Logistic regression analyses were selected to predict the relationship between sACE2 activity and biomedical factors. Differences were considered significant when *P* < 0.05. Receiver operating characteristic (ROC) curve analyses were used to evaluate the diagnostic performance (and optimal threshold) of sACE2 activity and NT-proBNP levels.

**Table 1:** Baseline characteristics of the study population

	Total cohort	NF-LG AS	NF-HG AS	LF-HG AS	LF-LG AS	PLF-LG AS
	*n* = 175 (100%)	*n* = 33 (19%)	*n* = 67 (38%)	*n* = 34 (19%)	*n* = 31 (18%)	*n* = 10 (6%)
Demographics						
Age (yrs)	76(70–81)	76(66.5–79)	76(71–82)	78(74–83)	76(70–81)	68.5(65–75)
Male	97(55)	16(49)	31(46)	18(53)	25(81)	7(70)
BSA (kg/m^2^)	1.9(1.8–2.1)	1.9(1.8–2.1)	1.8(1.8–2.0)	1.9(1.8–2.1)	2.0(1.9–2.2)	2.2(2.0–2.3)
SBP (Hgmm)	130 ± 22	138 ± 20	136 ± 23	124 ± 18	121 ± 21	126 ± 15
DBP (Hgmm)	78 ± 12	78 ± 9	81 ± 14	74 ± 12	76 ± 10	76 ± 9
HR (n/min)	72(65–85)	70(62–88)	70(64–80)	75(65–86)	80(70–100)	70(68–79)
Echocardiography						
AVA (cm^2^)	0.7(0.6–0.8)	0.8(0.7–0.9)	0.7(0.6–0.8)	0.5(0.45–0.6)	0.8(0.7–0.9)	0.8(0.7–0.85)
Mean gr. (Hgmm)	43(32–52)	31(28.5–35)	51(45–65)	47.5(45–62)	30(22–33.5)	33(30.5–35)
Peak gr. (Hgmm)	67(53–82)	53(44.5–57.5)	81(71–96)	78.5(68–86)	50(34–54)	52.5(47–56)
Peak velocity (m/sec)	4(3.6–4.5)	3.6(3.4–3.8)	4.5(4.1–4.9)	4.4(4.1–4.6)	3.5(2.9–3.7)	3.7(3.6–3.8)
LVEF (%)	54(43–58)	58(55–60)	56(55–60)	43(38–46)	32.5(30–38)	55(51–58)
LVESD (mm)	35(30–40)	30(28–35.5)	32(28–35)	40(36–43.5)	46(39.5–54)	33(28–39)
LVEDD (mm)	54(50–58)	50(48–54)	52(46–57)	56(53.5–60)	58(56.5–66)	52(50–59)
IVSth(mm)	13(12–14)	13(12–14)	13.5(13–15)	14(12.5–15.5)	12.3(11–14)	12.5(11.8–14.6)
LAD (mm)*	45 ± 7	41 ± 5	44 ± 6,5	47 ± 5	48 ± 7	47 ± 8
TAPSE (mm)*	21 ± 5	24 ± 4	22 ± 4	19.5 ± 4	18 ± 5	19.5 ± 4
sPAP (Hgmm)*	40.5 ± 13	39 ± 12	39 ± 12	44 ± 14	40.5 ± 15	44 ± 7
Cardiovascular comorbidities						
Hypertension	165(94)	31(94)	62(93)	32(94)	31(100)	9(90)
Diabetes mellitus	61(35)	12(36)	20(30)	14(41)	11(36)	4(40)
Hyperlipidaemia	110(63)	21(64)	40(60)	21(62)	21(68)	7(70)
IHD	60(34)	8(24)	16(24)	10(29)	21(68)	5(50)
PCI	46(26)	7(21)	14(21)	6(18)	17(55)	2(20)
CABG	15(10)	2(6)	2(3)	3(9)	5(16)	3(30)
AF in history	62(35)	7(21)	16(24)	15(44)	17(55)	7(70)
Ongoing AF	36(21)	3(9)	9(13)	6(18)	12(39)	6(60)
Medications						
ACEI/ARB	130(74)	28(85)	49(73)	23(68)	23(74)	7(70)
Beta-blockers	131(75)	23(70)	49(73)	26(77)	24(77)	9(90)
Aldosterone antagonist	68(39)	6(18)	14(20)	19(56)	25(80)	4(40)
Statins	107(61)	18(55)	39(58)	21(62)	22(71)	7(70)
Diuretics	134(77)	18(55)	46(69)	31(91)	29(94)	10(100)
Biochemical markers						
Hgb (g/l)	130 ± 18	134 ± 17	128 ± 20	126 ± 18	136 ± 15,5	135 ± 12
GFR (ml/min)	64(52–83)	62(51–83.5)	70(54–86)	56(46–69)	71(40–80)	79(57–90)
BUN (mmol/l)	7.3(5,8–10)	7(5.5–9.3)	6.9(5.7–9.4)	8.2(5.8–12.5)	9.9(7.5–13.7)	6.8(5.5–8.4)
Creatinine (qmol/l)	86(73–108)	83(70–110)	82(70–101)	96.5(82–115)	90(78–116)	81(57–115)
CRP (mg/l)	2.5(1.2–6.6)	1.9(1–4.2)	1.7(1–5)	4(1.5–11)	4.4(1.5–11)	3.1(1.7–9.1)

Values are mean ± SD, n(%), or median(IQR). *More than 10% of data not available. *AS*, Aortic Stenosis; *NF*, Normal-Flow; *LF*, Low-Flow; *LG*, Low-Gradient; *NG*, Normal-Gradient; *LV*, Left Ventricle; *BSA*, Body Surface Area; *SBP*, Systolic Blood Pressure; *DBP*, Diastolic Blood Pressure; *HR*, Heart Rate; *AVA*, Aortic Valve Area; *Gr.*, Gradient; *LVEF*, Left Ventricular Ejection Fraction; *LVESD*, Left Ventricular End-Systolic Diameter; *LVEDD*, Left Ventricular End-Diastolic Diameter; *IVSth*, Interventricular Septum thickness; *LAD*, Left Atrium Diameter; *TAPSE*, Tricuspid Annular Plane Systolic Excursion; *sPAP*, systolic Pulmonary Artery Pressure; *PTCA*, Percutaneous Coronary Intervention; *CABG*, Coronary Artery Bypass Grafting; *IHD*, Ischemic Heart Disease; *AF*, Atrial Fibrillation; *ACEI*, angiotensin-converting enzyme inhibitor; *ARB*, angiotensin receptor blocker; *Hgb*, Hemoglobin; *GFR*, Glomerular Filtration Rate; *BUN*, Blood Urea Nitrogen; *CRP*, C-Reactive Protein

## Results

### Baseline characteristics of study groups

According to the AS grading classification, 19% (*n* = 33) NF-LG, 38% (*n* = 67) NF-HG, 19% (*n* = 34) LF-HG, 18% (*n* = 31) LF-LG and 6% (*n* = 10) PLF-LG AS were found among the recruited patients. The baseline characteristics (i.e. demographics, comorbidities, medications, echocardiographic parameteres, biochemical markers) for all cohort and categorized groups are presented in Table 1. There were no significant differences between pharmacological therapy among the patient groups. Patients with LF-LG and PLF-LG AS were more likely to be men and have a history of more frequent cardiovascular diseases (CVD). Patients with PLF-LG AS were more likely to have ongoing atrial fibrillation or have it in history.

### sACE2 activity and NT-proBNP levels gradually increase with the progression of severe AS

**Fig. 1:**
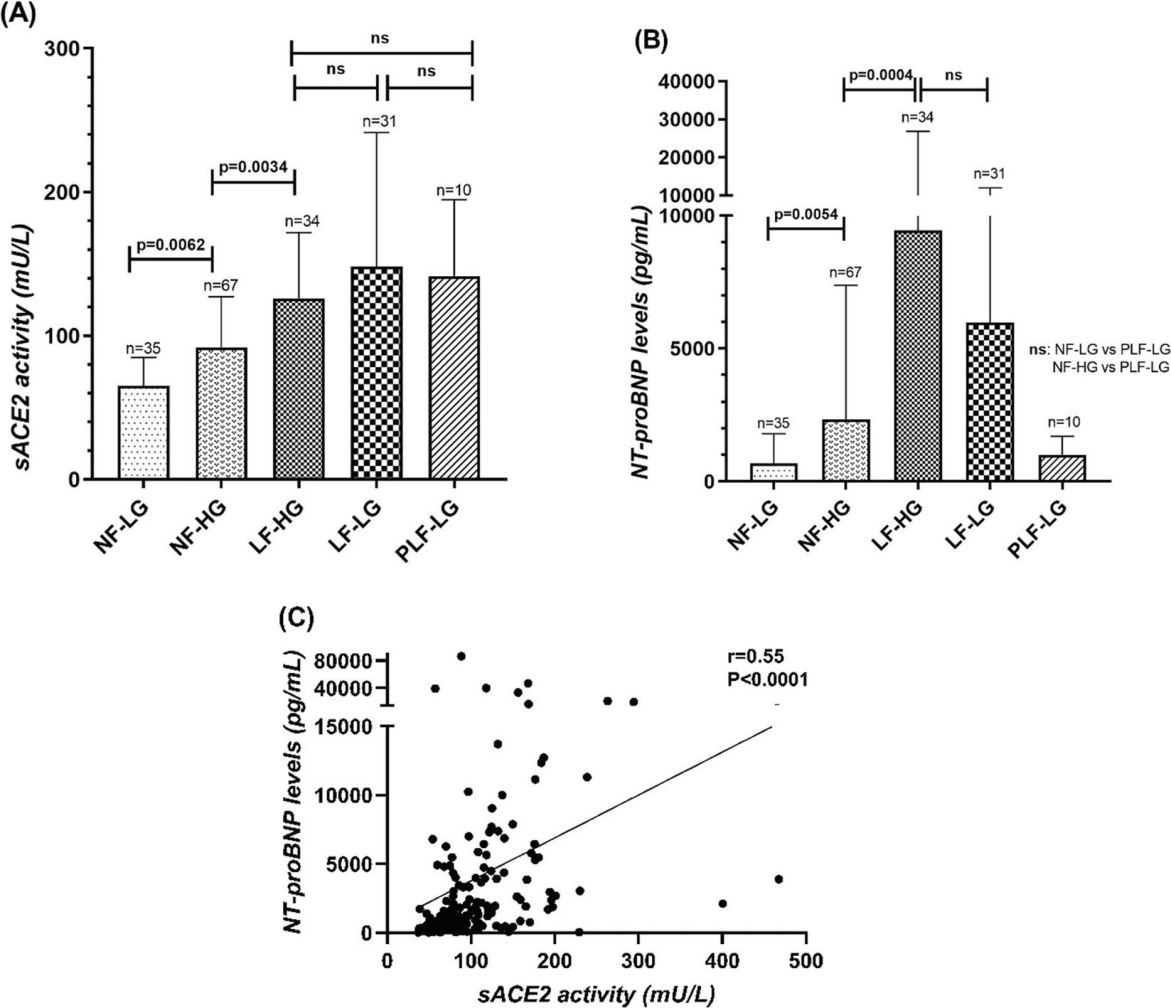
(**A**-**C**), sACE2 activity and NT-proBNP levels gradually increase with the progression of severe AS. (**A**) sACE2 activity was the lowest in NF-LG AS and gradually increased along the patient subgroups, representing the progression of severe AS paralell with the deterioration of haemodynamic parameters. sACE2 activity did not differ significantly between LF-HG and LF-LG AS groups and in patients with PLF-LG AS was similarly high as in the LF groups. (**B**) NT-proBNP levels also showed a gradual increase during the progression of AS, but in patients with PLF-LG AS remained as low as those measured in the NF AS groups. (**C**) A moderate correlation was found between sACE2 and NT-proBNP levels. Columns show the mean and SEM. Statisical significance was tested by the non-parametric Kruskal–Wallis test among the groups. ns = not significant; AS = Aortic Stenosis; NF = Normal-Flow; LF = Low-Flow; LG = Low-Gradient; NG = Normal-Gradient

sACE2 activity was the lowest in NF-LG AS (65.0 ± 3.5 U/ml) and gradually increased along the patient subgroups (NF-HG AS: 92.1 ± 4.3 U/mL, LF-HG AS: 126 ± 7.9 U/mL, LF-LG AS: 148.1 ± 16.8 U/mL; *P* < 0,05 between NF-LG AS vs. NF-HG AS, NF-HG AS vs. LF-HG AS, respectively), representing the progression of severe AS parallel with the deterioration of haemodynamic parameters (Fig. 1A). There was no significant difference in sACE2 activity between LF-HG and LF-LG AS groups, and PLF-LG AS was similar to the LF groups (141.6 ± 16.8 U/ml, Fig. 1A).

NT-proBNP levels also showed a gradual increase across the NF-LG, NF-HG and LF-HG AS patient groups (NF-LG AS: 687 ± 193 pg/mL, NF-HG AS: 2341 ± 615 pg/mL, LF-HG AS: 9434 ± 2993 pg/mL, LF-LG AS 5966 ± 1076 pg/mL, *P* < 0.05 between NF-LG vs. NF-HG and NF-HG vs. LF-HG), but NT-proBNP levels in patients with PLF-LG AS (1010 ± 218 pg/mL) remained similarly low as those measured in the NF-LG and NF-HG AS groups (Fig. 1B). A moderate positive correlation was found between sACE2 and NT-proBNP levels (*r* = 0.55, *P* < 0.0001, Fig. 1C).

### Correlations between echocardiographic parameters with sACE2 activity and NT-proBNP levels

**Fig. 2:**
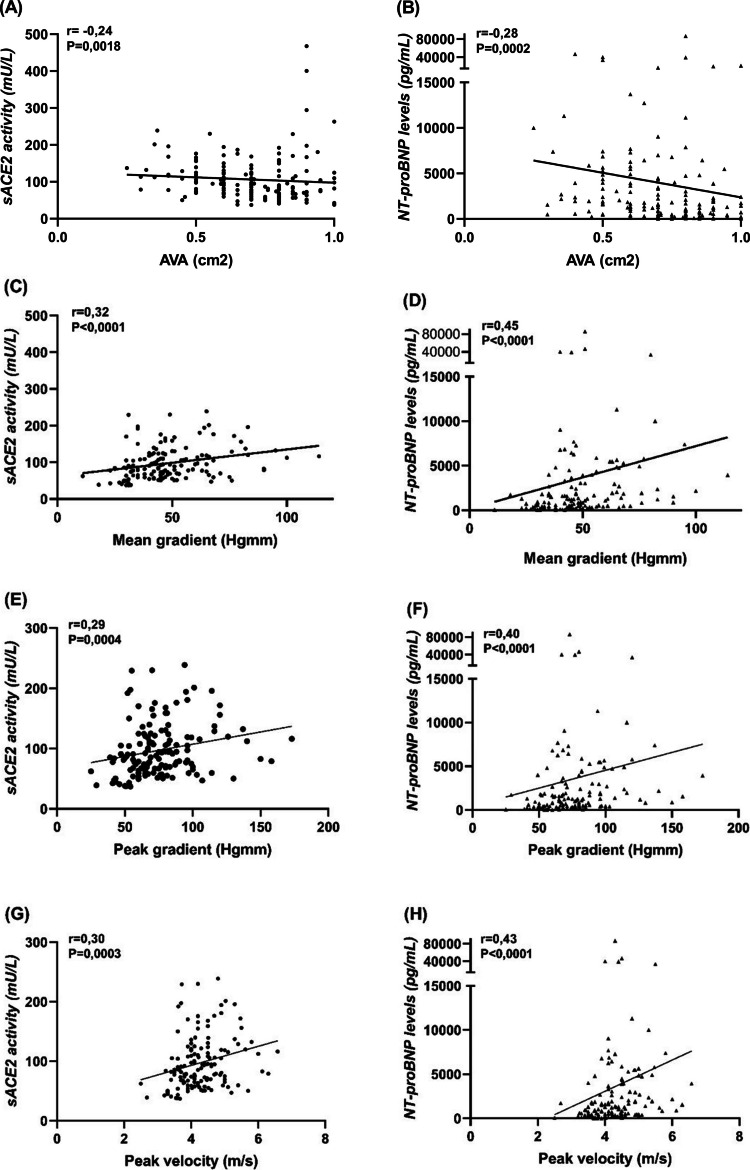
(**A**-**H**), sACE2 activity and NT-proBNP levels correlated with the severity of aortic stenosis. Correlation of sACE2 activity and NT-proBNP were tested with AVA (**A-B**), mean gradient (**C-D**), peak gradient (**E-F**) and peak velocity (**G-H**). The data tables show the parameters of the Spearman’s rank correlation (the coefficient rho is represented by the r value). AVA = Aortic Valve Area

**Fig. 3:**
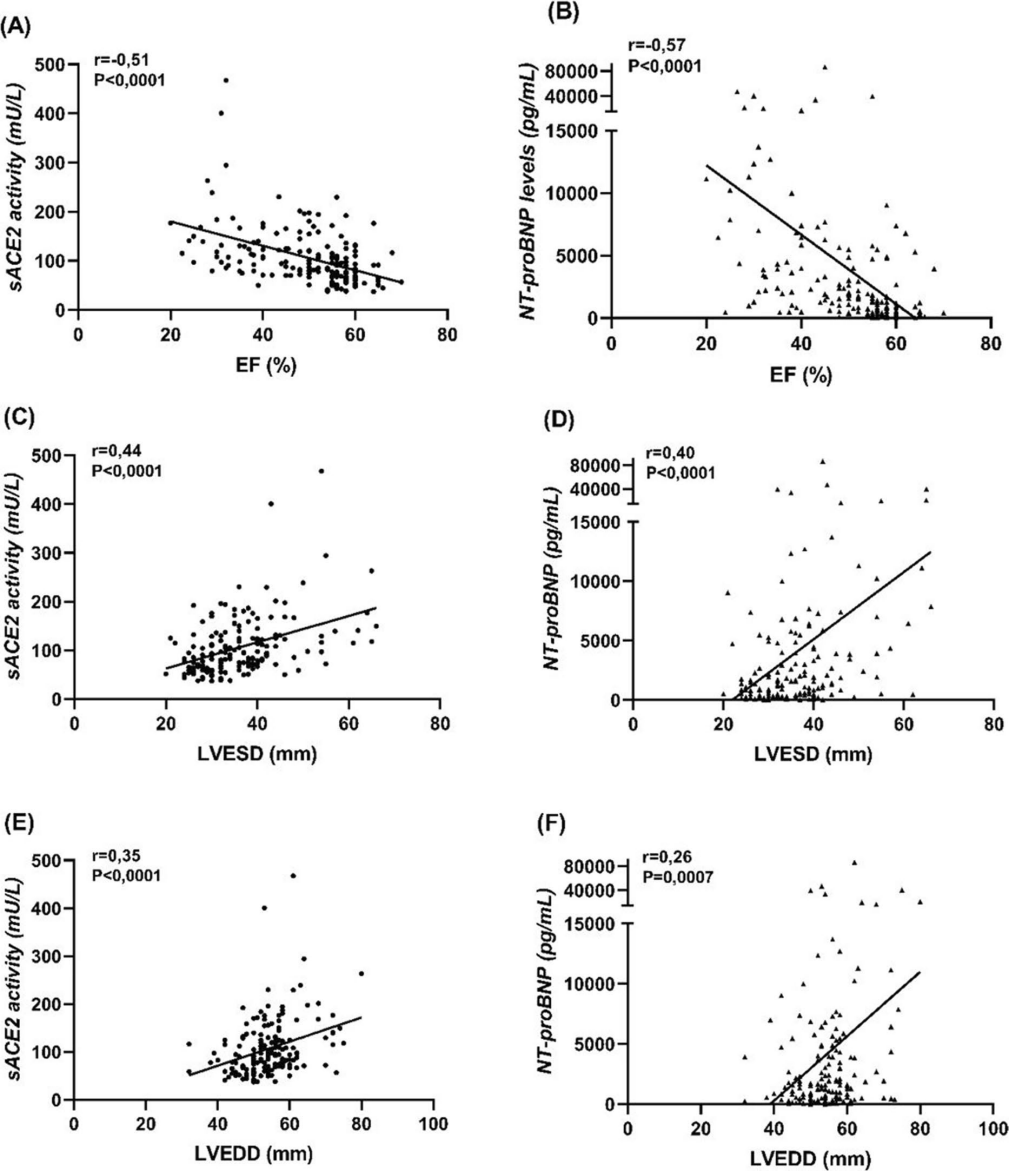
(**A**-**F**), Both biomarkers correlated with the left ventricular systolic function. For both biomarkers a negative correlation was confirmed with the left ventricular ejection fraction (**A**-**B**), and hence a positive correlation with left ventricular end-systolic (**C**-**D**) and end-diastolic (**E**-**F**) diameters. The data tables show the parameters of the Spearman’s rank correlation (the coefficient rho is represented by the r value). EF = Ejection Fraction; LVESD = Left Ventricular End-Systolic Diameter; LVEDD = Left Ventricular End-Diastolic Diameter

**Fig. 4:**
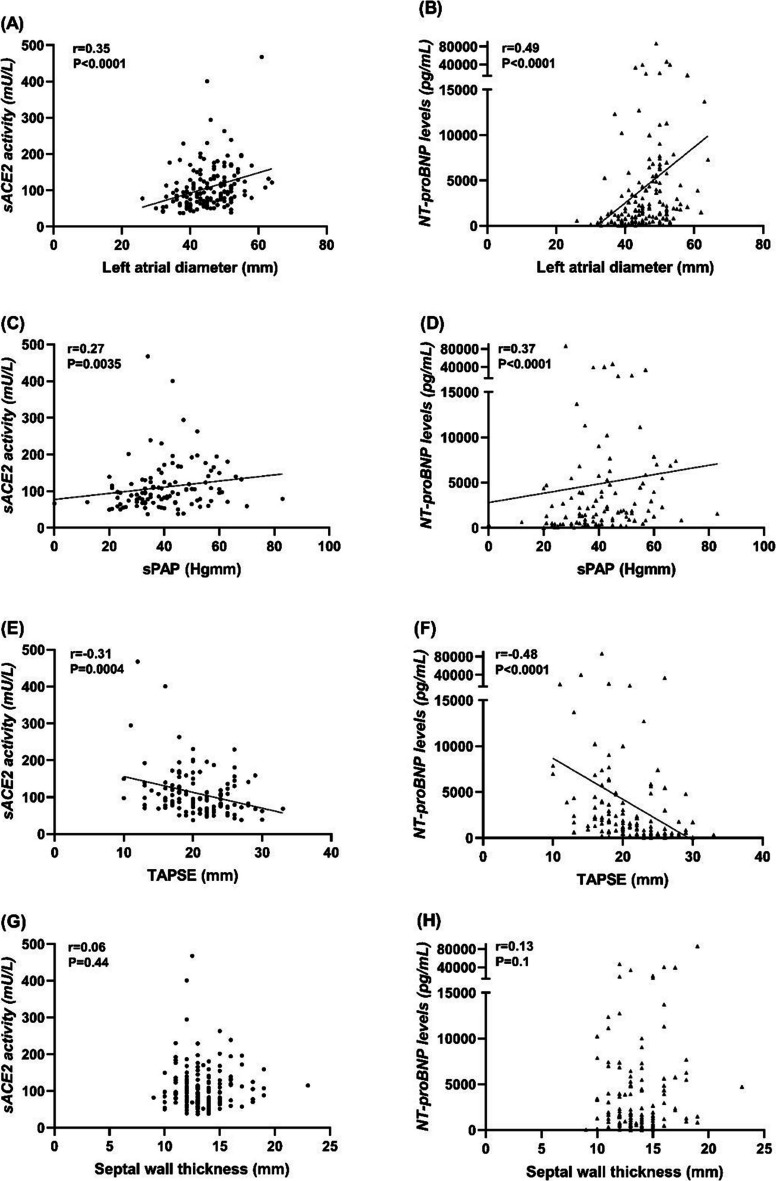
(**A**-**F**), sACE2 activity and NT-proBNP similarly correlate with echocardiographic parameters. sACE2 activity and NT-proBNP levels positively correlated with the left atrial diameter (**A**-**B**), the sPAP (**C**-**D**) and negatively with the TAPSE (**E-F**). No correlations were found between these biomarkers and septal wall thickness (**G**-**H**). The data tables show the parameters of the Spearman’s rank correlation (the coefficient rho is represented by the r value). sPAP = Systolic Pulmonary Arterial Pressure; TAPSE = Tricuspid Annular Plane Systolic Excursion

Both sACE2 and NT-proBNP correlated negatively with AVA (*r* = −0.24; *P* = 0.0018 and *r* = −0.28; *P* = 0.0002, Fig. 2A and B) and except of LF-LG AS group further moderate positive correlations were confirmed with haemodynamic parameters: with the AV mean gradient (*r* = 0.32; *P* < 0.0001 and *r* = 0.45; *P* < 0.0001, Fig. 2C and D), the AV peak gradient (*r* = 0.29; *P* = 0.0004 and *r* = 0.40; *P* < 0.0001, Fig. 2E and F) and the AV peak velocity (*r* = 0.30; *P* = 0.0003 and *r* = 0.43; *P* < 0.0001, Fig. 2G and H)*.* A moderate negative correlation was found for both biomarkers with the LVEF (*r* = −0.51; *P* < 0.0001 and *r* = −0.57; *P* < 0.0001, Fig. 3A and B), contrasted by positive correlations with LV end-systolic diameters (*r* = 0.44, *P* < 0.0001 and *r* = 0.4, *P* < 0.0001, Fig. 3C and D) and LV end-diastolic diameters (*r* = 0.35, *P* < 0.0001 and *r* = 0.26, *P* = 0.0007, Fig. 3E-F). In addition, sACE2 activity and NT-proBNP levels positively correlated with the left atrial diameter (*r* = 0.35, *P* < 0.0001 and *r* = 0.49, *P* < 0.0001, Fig. 4A and B), the systolic pulmonary arterial pressure (*r* = 0.27, *P* = 0.0035 and *r* = 0.37, *P* < 0.0001, Fig. 4C and D) and negatively with the tricuspid annular plane systolic excursion (*r* = −0.31, *P* = 0.0004 and *r* = −0,48, *P* < 0.0001, Fig. 4E and F). No correlations were found between these biomarkers and septal wall thickness (*r* = 0.06, *P* = 0.44 and *r* = 0.13, *P* = 0.1, Fig. 4G and H).

Regarding the AS independent (confounding) parameters, NT-proBNP levels, but not sACE2 activity correlated with age, Hgb, BUN, eGFR and diastolic blood pressure. Both biomarkers were affected by systolic blood pressure, CRP and creatinine, although sACE2 activity was less altered by these parameters (Table 2).

**Table 2:** Non-AS related parameters that affected the sACE2 activity and NT-proBNP levels

	Total cohort	sACE2 activity (P)	NT-proBNP levels (P)
Age (yrs)	76(70–81)	ns	**
Systolic Blood Pressure (Hgmm)	130 ± 22	**	***
Diastolic Blood Pressure (Hgmm)	78 ± 12	ns	*
BUN	7.3(5.8–10)	ns	***
Creatinine	86(73–108)	*	****
GFR	64(52–83)	ns	****
Hgb	130 ± 18	ns	**
CRP	2.5(1.2–6.6)	***	****

The data table show the level of significancy (*P*).* = < 0,05; ** = < 0,005; *** = < 0,005;**** = < 0,0001; *ns*, not significant. Values are mean ± SD, n(%), or median(IQR)

### The clinical applicability of sACE2 in patients with severe AS

ROC analysis was used to evaulate the predictive value of sACE2 activity for subclinical dysfunction in NF AS (i.e. NF-LG and NF-HG) in the light of LF AS (i.e. LF-HG, LF-LG, PLF-LG AS) patients. We studied three subgroup analysis for sACE2 activity and NT-proBNP levels to predict the transition from compensated to decompensated LV function: NF AS vs. LF AS, NF-LG AS vs. LF AS and NF-HG AS vs. LF AS.

**Fig. 5:**
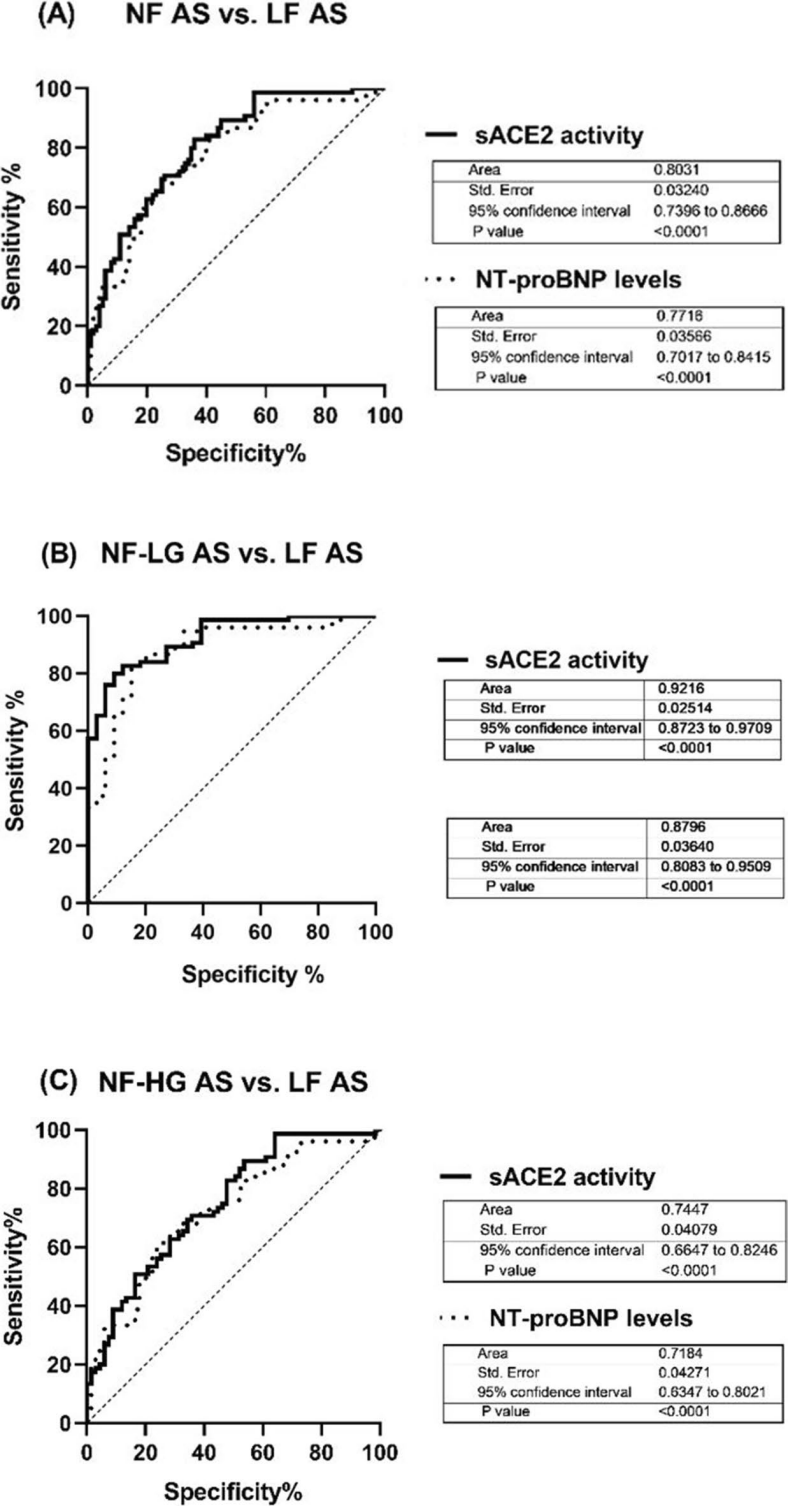
(**A**-**C**), The clinical applicability of sACE2 in patients with severe AS. Receiver operating characteristic (ROC) analysis was used to evaulate the predictive value of sACE2 activity and NT-proBNP levels for subclinical dysfunction in NF AS (i.e. NF-LG and NF-HG) in the light of LF AS (i.e. LF-HG, LF-LG, PLF-LG AS) patients

Area under the ROC curve (AUC) values for sACE2 activity and NT-proBNP levels were 0.80 vs.0.77, 0.92 vs.0.88, 0.75 vs.0.72, respectively. The highest optimal sensitivity while maintaining the highest specificity was 98.67%. At this level of sensitivity, the specificity levels for sACE2 and NT-proBNP were 44% vs. 6%, 60.6% vs. 15.2% and 35.8% vs. 1.5% respectively (Fig. 5A-F, Table 3).

**Table 3:** ROC curves showing the sensitivity and specificity of sACE2 and NT-proBNP

	sACE2 activity				NT-proBNP levels			
	Cutpoint	Sensitivity	Specificity	AUC	Cutpoint	Sensitivity	Specificity	AUC
NF AS vs. LF AS	70,01	98,67%	44%	0,80	74,02	98,67%	6%	0,77
NF-LG AS vs. LF AS	69,11	98,67%	60,6%	0,92	72,33	98,67%	15,2%	0,88
NF-HG AS vs. LF AS	70,01	98,67%	35,8%	0,75	74,02	98,67%	1,5%	0,72

The data table show the sensitivity and specificity of sACE2 and NT-proBNP for the presence of subclinical LV dysfunction. *AUC* Area under the curve

## Discussion

The pressure overload in AS triggers a hypertrophic response that maintains the left ventricular performance for many years. However, at later stages of AS this compensation becomes insufficient leading to heart failure. This process of adverse remodeling is complex, but is closely related to the development of myocyte injury, cell death and myocardial fibrosis [23]. These changes are regulated by several factors, including the RAS [24]. The connection between ACE2 and fibrosis in AS was strongly confirmed in a recent study [25]. Ramchand et al. described that in AS patients, myocardial ACE2 gene expression was reduced, reduced tissue levels were associated with elevated plasma ACE2 activity, and those with the highest plasma ACE2 activity had more severe myocardial fibrosis. Further, increased plasma ACE2 activity independently predicted mortality.

### The role of sACE2 activity in the assessment of severity of AS

The key finding of this study was that the sACE2 activity gradually increases with the progression of severe AS parallel with the deterioration of haemodynamic parameters. In contrast to previous studies [18, 25], here a positive correlation was confirmed between sACE2 activity and AVA and (apart from the LF-LG group), with mean and peak pressure gradients above the aortic valve. In line with earlier observations, sACE2 activity negatively correlated with LVEF**.** Interestingly, sACE2 activity in NF-LG AS patients was 1.5-fold higher than in patients with reduced EF without AS [15, 26], and further increased in the NF-HG AS group despite no significant deterioration in LVEF. Important to note that performing subgroup analysis, sACE2 activity did not correlate with LVEF in the different AS patient groups.

Based on our results, no single factor influences the elevation of sACE2 activity as a valvular-specific cause. The gradual increase in sACE2 activity reflects the progressive worsening of myocardial structural abnormalities, as shown by correlations with left atrial diameter, sPAP, TAPSE, LV diameters and LVEF. Interestingly, no correlation was found with LV septal thickness, the first maladaptive response to chronic pressure overload. These results are consistent with a previous study [18].

We found that NT-proBNP levels showed similar tendencies as the sACE2 activity, in line with a previous observation(7). This suggests that both biomarkers are affected by the progression of the disease, supporting the growing evidence that sACE2 activity is a reliable marker of myocardial remodeling and fibrotic damage.

### Predictive value of cardiac biomarkers in the risk stratification of AS

The timing of intervention for severe AS patients is clearly defined [3], however, pathophysiological considerations and accumulating clinical evidence suggest a potential benefit of earlier intervention for patients with AS than the current guidelines [2, 5, 27–29]. The TAVR-UNLOAD (NCT0266145) and PROGRESS (NCT04889872) randomized trials are currently testing the hypothesis of earlier intervention in moderate AS and risk features [30]. These results might bring the need to re-evaluate AS severity assessment. A multiparametric approach, including biomarkers that can reflect early LV remodeling, could optimize the timing of the procedure.

The predictive value of NT-proBNP in severe AS is well established [3, 7, 31]. In our study, we found that sACE2 activity was a stronger predictor for subclinical dysfunction than NT-proBNP levels. Furthermore, in NF-LG AS specificity was considerably higher compared to NF-HG AS, supporting the fact that maladaptive myocardial changes are less expressed in NF-LG AS and in line with the natural history of AS, in NF-HG AS indicates a greater number of patients with severe structural changes who could benefit from earlier AVR.

Our findings are underscore the view that paradoxical AS is a disease with pronounced myocardial remodeling [32] as sACE2 activity was as high as in LF AS groups unlike NT-proBNP levels. Data support that the risk of intervention in PLF-LG AS is about 3.5 times higher than in NF AS [33] and TAVR confers significant benefit compared to a conservative approach [34] suggesting that identification of this pattern of AS is essential.

These results support the hypothesis that measurement of sACE2 activity might aid in identifying high risk patients with subclinical LV dysfunction and provides incremental value over NT-proBNP.

### Factors affecting biomarkers

Biomarker levels can be affected by various clinical factors, limiting their clinical applicability. In our study, males had significantly higher sACE2 activity than females, confirming earlier reports [18, 25]. Despite this gender difference, sACE2 activity increased in parallel with myocardial dysfunction in both genders. Earlier reports also indicated that sACE2 activity increased with age in hypertensive patients [15, 18], however, our results did not confirm such age dependency.

There were no effects of comorbidities or cardiovascular diseases (i.e., diabetes, dyslipidemia, ischemic heart disease, previous PCI or coronary bypass grafting) on sACE2 activity in the different groups of severe AS, except for ongoing atrial fibrillation, which had a slight effect only in the HG-NF AS group. This suggests that the increase in sACE2 activity parallel with myocardial abnormalities is robust enough to diminish the prominent effect of atrial fibrillation on basal sACE2 activity [19].

Furthermore, we confirmed that the increase in sACE2 activity was not independent of impaired kidney function. We observed a moderate positive correlation between creatinine and sACE2 activity in the NF group, but no correlation was detected with urea or calculated GFR.

In contrast, in our study, NT-proBNP was affected by numerous volume-mediated comorbidities, leading to confounding states: ongoing atrial fibrillation had a significant effect on NF-LG, NF-HG, and PLFLG AS. We confirmed a strong positive correlation between kidney function (creatinine, urea, and GFR) and NT-proBNP levels. Additionally, patients on dialysis had extremely high, outlier values, consistent with previous knowledge [35].

We also examined the relationship between NT-proBNP and EF in subgroups and found a positive correlation in all groups, above the overall cohort correlation. This correlation with sACE2 activity was absent in subgroups. Overall, based on our data, NT-proBNP levels reflect worsening volume overload during the progression of severe AS rather than malignant myocardial changes.

### Study limitations

This study has several limitations that should be considered when interpreting the findings. First, it was conducted at a single center, on a Caucasian population, which may limit the generalizability of the results to broader populations. Second, although the study included 175 patients, this sample size may still be considered relatively small, particularly when analyzing subgroups, such as those with PLF-LG AS. Additionally, the cross-sectional design of the study restricts the ability to draw causal inferences regarding the relationship between sACE2 activity and the stages of AS. Longitudinal studies would be necessary to establish temporal relationships and better understand the progression of the disease. While the study controlled for some known variables, there may be unmeasured confounding factors, such as comorbidities, medications, and lifestyle factors, that could affect biomarker levels and their relationship with AS severity. The lack of long-term follow-up data on patient outcomes related to sACE2 activity is another limitation. Future studies should assess how changes in sACE2 levels over time correlate with clinical outcomes. Lastly, potential selection bias exists, as patients included in the study were those undergoing preoperative evaluation or valve intervention, which may not fully represent the entire population of patients with severe AS, particularly asymptomatic individuals. Additionally, the study population may lack diversity in terms of ethnicity and demographics, which could limit the applicability of the findings to other populations.

## Conclusions

In this study, we demonstrated that sACE2 activity is significantly associated with the different flow-grade pattern of severe AS. Our findings indicate that sACE2 activity increase progressively with the natural history of severe AS, suggesting its potential role as an objective biomarker for assessing LV dysfunction and disease progression, offering incremental value over traditional biomarkers such as NT-proBNP.

The ability of sACE2 to correlate with echocardiographic parameters further underscores its utility in clinical practice, particularly in guiding the timing of AVR and identifying patients at risk for adverse outcomes. Given the limitations of existing biomarkers, sACE2 presents a promising avenue for enhancing risk stratification and improving patient management in AS.

## Data Availability

The data analyzed and presented in this study are available from the corresponding author on request.
